# Headache in Patients With Non‐Thrombotic Internal Jugular Vein Stenosis: Clinical Characteristics and Associated Risk Factors in a Retrospective Study of 283 Cases

**DOI:** 10.1111/cns.70594

**Published:** 2025-09-04

**Authors:** Guangyu Han, Shuling Wan, Xunming Ji, Ran Meng, Da Zhou

**Affiliations:** ^1^ Department of Neurology, Xuanwu Hospital Capital Medical University Beijing China; ^2^ Advanced Center of Stroke Beijing Institute for Brain Disorders Beijing China; ^3^ National Center for Neurological Disorders, Xuanwu Hospital Capital Medical University Beijing China

**Keywords:** clinical characteristics, headache, internal jugular vein stenosis, logistic regression analysis, risk factors

## Abstract

**Aims:**

This study aimed to characterize the clinical features of headache in patients with non‐thrombotic internal jugular vein stenosis (IJVS) and to identify associated risk factors.

**Methods:**

This retrospective study consecutively enrolled patients with imaging‐confirmed non‐thrombotic IJVS from January 2021 through July 2024. Participants were divided into IJVS‐headache and IJVS‐without‐headache groups based on clinical symptoms. Demographic, clinical, neuroimaging, and treatment data were reviewed in detail. Univariate and multivariate logistic regression analyses were performed to determine risk factors for headache.

**Results:**

Among 283 eligible patients (median age: 51 years in the IJVS‐headache group vs. 56 years in the IJVS‐without‐headache group, *p* < 0.001), 65.02% reported headache. Most headaches were chronic (82.07%), generalized (85.87%), and moderate in intensity (53.26%), with notable daily life impact (57.61%). Univariate analysis showed that headache was significantly associated with visual disturbances (*p* = 0.010), elevated cerebrospinal fluid opening pressure (*p* < 0.001), high jugular bulb (*p* = 0.007), and severe scalp vein dilation (*p* < 0.001), but inversely associated with severe vertebral vein expansion (*p* < 0.001). Multivariate regression revealed that high jugular bulb (OR = 3.144, 95% CI: 1.083–9.123, *p* = 0.035), severe scalp vein dilation (OR = 2.142, 95% CI: 1.068–4.294, *p* = 0.032), and protein C or S deficiency (OR = 5.984, 95% CI: 1.196–29.928, *p* = 0.029) were independent risk factors, whereas severe vertebral vein expansion was protective (OR = 0.184, 95% CI: 0.092–0.366, *p* < 0.001).

**Conclusions:**

Headache represents a prevalent and often disabling symptom in non‐thrombotic IJVS, underpinned by distinctive vascular and hematologic profiles. Identification of high‐risk patients based on neuroimaging and thrombophilia screening may facilitate personalized interventions and improve symptom control.

## Graphical abstract

Approximately two‐thirds of non‐thrombotic IJVS patients experienced headache, generally moderate, chronic, and diffuse. Identifying clinical and neuroimaging risk factors of headache may help guide individualized management strategies to reduce headache occurrence.

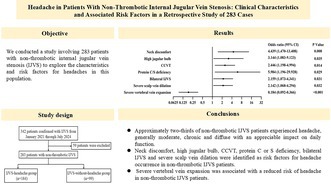

**Abbreviations**
CCVT: cerebral cortical venous thrombosis
CDFI: color Doppler flow imaging
CE‐MRV: contrast‐enhanced magnetic resonance venography
CI: confidence interval
CSF: cerebrospinal fluid
CTV: computerized tomography venography
CV: catheter venography
CVSS: cerebral venous sinus stenosis
CVST: cerebral venous sinus thrombosis
HIT‐6: Headache Impact Test‐6
IJV: internal jugular vein
IJVS: internal jugular vein stenosis
IJVT: internal jugular vein thrombosis
MRBTI: magnetic resonance black‐blood thrombus imaging
ONSD: optic nerve sheath diameter
OR: odds ratio
VAS: visual analog scale

## 1 Introduction

Internal jugular vein stenosis (IJVS), characterized by ≥ 50% luminal narrowing of the internal jugular vein (IJV), has gained increasing recognition as a potential contributor to a spectrum of neurological symptoms [1, 2, 3, 4, 5, 6]. While traditionally underappreciated, non‐thrombotic IJVS has been increasingly implicated in a range of nonspecific clinical presentations, including headache, tinnitus, head noises, visual disturbances, dizziness, and sleep disorders [1, 2, 3, 4, 5, 6, 7]. Among these, headache is frequently reported but remains poorly characterized in terms of its clinical features, pathophysiological basis, and associated risk factors [1, 3, 4, 5, 8, 9].

Emerging evidence suggests that impaired cerebral venous outflow due to IJV narrowing may lead to intracranial venous hypertension, altered cerebrospinal fluid (CSF) dynamics, and venous ischemia, all of which may contribute to the development of headache [9, 10, 11, 12]. Despite its potential relevance, the role of headache in patients with non‐thrombotic IJVS has not been systematically explored, and its clinical correlates remain largely undefined.

To address this gap, we conducted a single‐center retrospective study of 283 patients diagnosed with non‐thrombotic IJVS. Our aim was to characterize the prevalence and features of headache in this population, identify independent risk factors, and provide insights into potential mechanisms. A better understanding of IJVS‐related headache may aid in timely recognition, appropriate workup, and individualized management strategies for affected patients.

## 2 Methods

### 2.1 Study Design and Patient Selection

**FIGURE 1:**
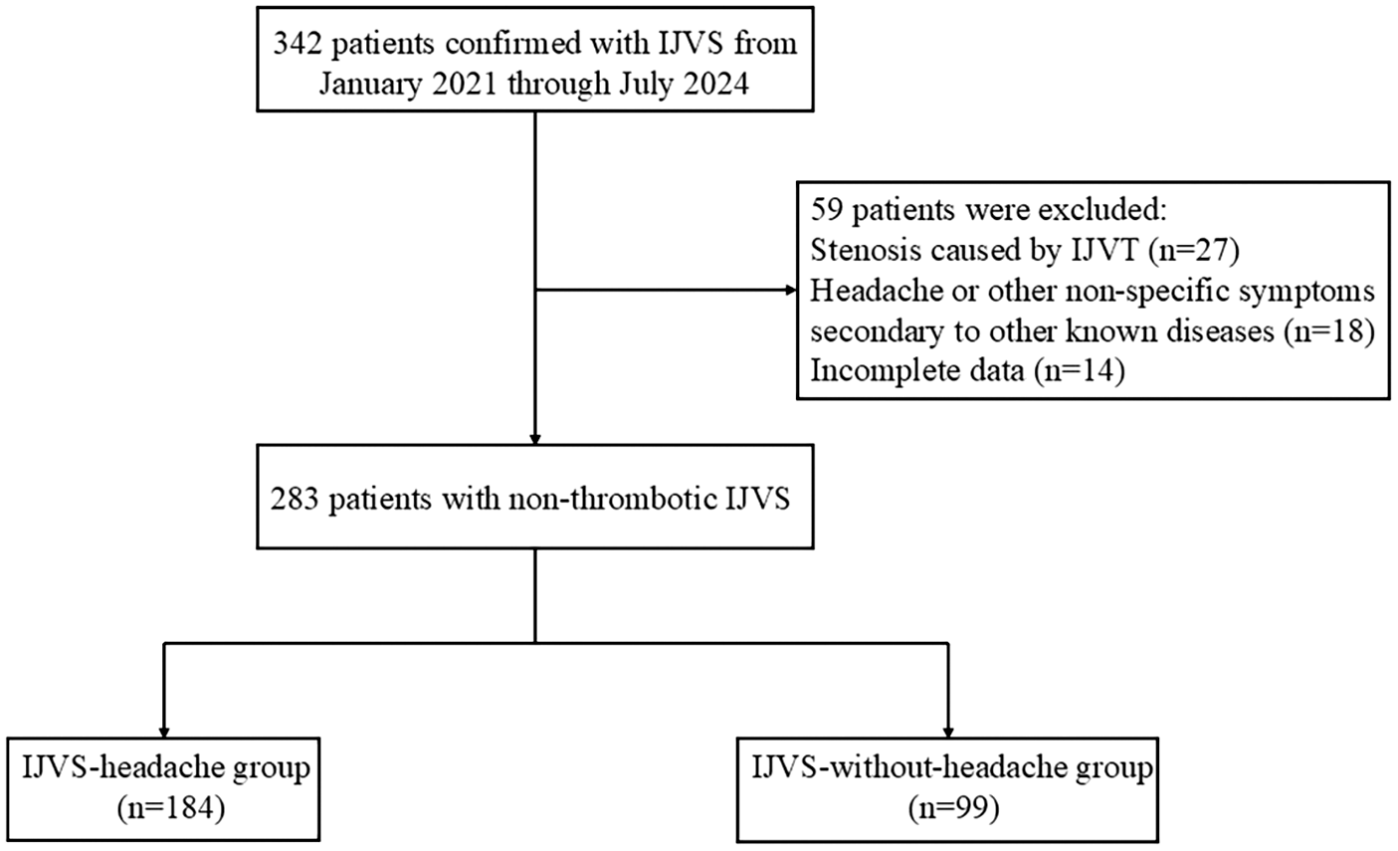
Flow chart of the study. IJVS, internal jugular venous stenosis; IJVT, internal jugular venous thrombosis.

This single‐center retrospective study was conducted at Xuanwu Hospital, Capital Medical University, between January 2021 and July 2024. A total of 283 patients diagnosed with non‐thrombotic IJVS were consecutively enrolled (Figure 1). The diagnosis of IJVS was established based on at least one imaging modality, including color Doppler flow imaging (CDFI), contrast‐enhanced magnetic resonance venography (CE‐MRV), computed tomography venography (CTV), or catheter venography (CV), demonstrating ≥ 50% stenosis of the IJV lumen relative to an adjacent normal segment.

Patients were eligible for inclusion if they met all of the following criteria: (1) Unilateral or bilateral IJVS confirmed by imaging; (2) Presence of abnormal venous collaterals visualized on CE‐MRV, CTV, or CV; (3) Presence of clinical symptoms consistent with cerebral venous outflow disturbance, such as headache, tinnitus, head noises, dizziness, visual disturbances, or sleep disorders; (4) No restriction on age or sex; and (5) Provision of signed informed consent. Patients with concomitant intracranial venous pathologies—including cerebral venous sinus thrombosis (CVST), cerebral cortical vein thrombosis (CCVT), or non‐thrombotic cerebral venous sinus stenosis (CVSS)—were also eligible, provided that IJVS was identified as the predominant lesion and no exclusion criteria were met.

Exclusion criteria were as follows: (1) Presence of internal jugular vein thrombosis (IJVT) confirmed by magnetic resonance black‐blood thrombus imaging (MRBTI); (2) Headache secondary to other intracranial or systemic conditions unrelated to the cerebral or jugular venous system (e.g., intracranial mass lesions, migraine, subarachnoid hemorrhage); (3) IJVS‐like symptoms (excluding headache) attributable to other well‐established diagnoses; and (4) Incomplete clinical or imaging data.

Following enrollment, patients were divided into two groups based on the presence or absence of headache: the IJVS‐headache group and the IJVS‐without‐headache group.

### 2.2 Clinical Data Collection and Evaluation

Demographic data and clinical characteristics were collected for all participants. Headache severity was graded using a visual analog scale (VAS) and classified as mild (1–3), moderate (4–6), or severe (7–10) [13]. Functional impairment of headache was evaluated using the Headache Impact Test‐6 (HIT‐6), stratified into four levels: little or no impact (≤ 49), some impact (50–55), substantial impact (56–59), and severe impact (60–78) [14].

Lumbar puncture was performed to measure the CSF opening pressure. Papilledema was evaluated using color fundus photography, while bilateral optic nerve sheath diameter (ONSD) was measured via transorbital ocular ultrasound.

Blood samples were collected to assess thrombophilia‐related markers, including protein C, protein S, antithrombin III, D‐dimer, and homocysteine. Additional testing for inflammatory markers and autoimmune panels (e.g., antiphospholipid antibodies) was performed when clinically indicated. Thrombophilic abnormalities were defined using standard diagnostic thresholds. Based on previous findings from our institution, hepatitis B virus (HBV) status was also recorded, with prior infection defined as negative hepatitis B surface antigen (HBsAg) and positive hepatitis B core antibody (anti‐HBc) and/or surface antibody (HBsAb) [1].

### 2.3 Neuroimaging Assessment

All patients underwent CE‐MRV and CTV to determine the laterality and extent of IJVS. In selected cases, CV was additionally performed for diagnostic confirmation or to guide endovascular treatment planning. Stenotic subtypes were classified by CDFI or CTV. Intraluminal stenosis was defined by the presence of malformed venous valves or mural thickening, while extraluminal compression was attributed to adjacent anatomical structures such as bony prominences or arteries.

The presence of a high‐positioned jugular bulb was assessed on CTV. Collateral venous pathways were evaluated using CE‐MRV, CTV, or CV and graded as either mild or severe, based on the maximum cross‐sectional area of vertebral venous collaterals relative to the proximal normal IJV. Mild collateralization was defined as < 25% of the normal IJV diameter, while severe collateralization was defined as ≥ 25% [2, 10]. Scalp vein dilation was categorized visually as absent, mild, or severe. Non‐thrombotic CVSS was confirmed by CE‐MRV.

Given that the IJV serves as the principal route for cerebral venous outflow, its stenosis can contribute to intracranial venous drainage disturbance and frequently coexists with other cerebral venous disorders [3, 4, 11]. Therefore, all patients underwent MRBTI to screen for coexisting CVST, CCVT, and ischemic or hemorrhagic venous infarctions.

All neuroimaging evaluations were independently reviewed by two senior neuroradiologists who were blinded to patient group assignments and clinical data. Any discrepancies in interpretation were resolved through discussion to reach consensus or, if needed, adjudicated by a third experienced neuroradiologist.

### 2.4 Statistical Analysis

All statistical analyses were performed using the Statistical Package for the Social Sciences (SPSS) version 28.0 program (IBM, USA). The normality of continuous variables was assessed using the Kolmogorov–Smirnov test. Variables following a normal distribution were reported as mean ± standard deviation (SD); otherwise, they were presented as median (interquartile range). Categorical variables were expressed as counts and percentages. Univariate logistic regression analyses were performed according to demographic data, clinical characteristics, and radiological features. Results were reported as odds ratios (OR) with 95% confidence intervals (CI) to quantify the strengths of associations between variables. Variables with *p*‐values < 0.1 in univariate logistic analysis were included in multivariate logistic regression models to determine independent predictors of headache. A two‐tailed *p*‐value < 0.05 was considered statistically significant.

## 3 Results

### 3.1 Demographic and Baseline Characteristics of IJVS Patients With and Without Headache

Of the 283 eligible non‐thrombotic IJVS patients, 184 (65.02%) suffered from headaches (IJVS‐headache group), while 99 (34.98%) did not (IJVS‐without‐headache group). Overall, the sample was balanced by sex (133 males and 150 females) with a mean body mass index (BMI) of 24.43 ± 3.46 kg/m^2^; neither sex nor BMI differed significantly between groups (*p* = 0.703 and *p* = 0.170, respectively). Patients in the IJVS‐headache group were younger, with a median age of 51 (37–62) years, compared to 56 (47–65) years in the IJVS‐without‐headache group (*p* < 0.001). The median time from symptom onset to hospital admission was 9.96 (3.00–24.00) months, similar in both groups (*p* = 0.895). Smoking and alcohol use were reported by 24.03% and 27.21% of all patients, respectively (Table 1).

**TABLE 1:** Clinical features in non‐thrombotic IJVS patients with or without headache.

Variables	All (*n* = 283)	IJVS‐headache group (*n* = 184)	IJVS‐without‐ headache group (*n* = 99)	Univariate analysis	
				*p* value	OR (95% CI)
Demographics					
Gender (female), *n* (%)	150 (53.00)	96 (52.17)	54 (54.55)	0.703	1.100 (0.674–1.796)
Age (years), median (IQR)	52 (40–63)	51 (37–62)	56 (47–65)	< 0.001^a^	0.970 (0.952–0.988)
BMI (kg/m^2^), mean ± SD	24.43 ± 3.46	24.64 ± 3.69	24.04 ± 2.95	0.170	1.052 (0.978–1.132)
Onset‐to‐door time (months), median (IQR)	9.96 (3.00–24.00)	9.96 (2.64–24.00)	12.00 (3.00–24.00)	0.895	0.999 (0.991–1.008)
Clinical manifestations, *n* (%)					
Sleep disturbances	146 (51.59)	96 (52.17)	50 (50.51)	0.789	1.069 (0.656–1.743)
Dizziness	136 (48.06)	89 (48.37)	47 (47.47)	0.886	1.037 (0.636–1.690)
Tinnitus	131 (46.29)	86 (46.74)	45 (45.45)	0.836	1.053 (0.645–1.719)
Head noise	116 (40.99)	67 (36.41)	49 (49.49)	0.034^a^	0.584 (0.356–0.959)
Visual disturbances	109 (38.52)	81 (44.02)	28 (28.28)	0.010^a^	1.994 (1.179–3.372)
Nausea or vomiting	81 (28.62)	60 (32.61)	21 (21.21)	0.045^a^	1.797 (1.014‐3.184)
Anxiety or depression	73 (25.80)	53 (28.80)	20 (20.20)	0.116	1.598 (0.890–2.869)
Visual decline	58 (20.49)	43 (23.37)	15 (15.15)	0.105	1.708 (0.894–3.261)
Hearing impairment	58 (20.49)	34 (18.48)	24 (24.24)	0.253	0.708 (0.392–1.280)
Subjective memory decline	47 (16.61)	30 (16.30)	17 (17.17)	0.852	0.940 (0.489–1.805)
Neck discomfort	42 (14.84)	33 (17.93)	9 (2.02)	0.050^b^	2.185 (1.000–4.776)
Limb numbness	9 (3.18)	7 (3.80)	2 (2.02)	0.422	1.918 (0.391–9.414)
Vertigo	6 (2.12)	4 (2.17)	2 (2.02)	0.932	1.078 (0.194–5.990)
Complications					
CSF opening pressure (mmH_2_O), median (IQR)	185.00 (155.00–230.00)	195.00 (160.00–243.75)	165.00 (145.00–200.00)	< 0.001^a^	1.010 (1.005–1.015)
Papilledema, *n* (%)	60 (21.20)	46 (25.00)	14 (14.14)	0.035^a^	2.024 (1.050–3.902)
ONSD (mm), median (IQR)					
Right	4.00 (3.60–4.50)	4.09 (3.66–4.60)	3.80 (3.50–4.30)	0.004^a^	1.634 (1.174–2.274)
Left	4.00 (3.56–4.40)	4.08 (3.70–4.50)	3.75 (3.45–4.25)	0.001^a^	1.778 (1.247–2.537)
Comorbidities, *n* (%)					
Hypertension	106 (37.46)	67 (36.41)	39 (39.39)	0.621	0.881 (0.533–1.456)
T2DM	32 (11.31)	19 (10.33)	13 (13.13)	0.478	0.762 (0.359–1.616)
Hyperlipidemia	179 (63.25)	119 (64.67)	60 (60.61)	0.499	1.190 (0.719–1.970)
Hyperhomocysteinemia	29 (10.25)	20 (10.87)	9 (9.09)	0.638	1.220 (0.533–2.790)
Hyperuricemia	32 (11.31)	21 (11.41)	11 (11.11)	0.939	1.031 (0.475–2.235)
Previous HBV infection	146 (51.59)	92 (50.00)	54 (54.55)	0.466	0.833 (0.511–1.360)
High jugular bulb	43 (15.19)	36 (19.56)	7 (7.07)	0.007^a^	3.197 (1.366–7.483)
Non‐thrombotic CVSS	159 (56.18)	112 (60.87)	47 (47.47)	0.031^a^	1.721 (1.051–2.818)
CCVT	144 (50.88)	111 (60.33)	33 (33.33)	< 0.001^a^	3.041 (1.823–5.073)
CVST	57 (20.14)	44 (23.91)	13 (13.13)	0.033^a^	2.079 (1.059–4.081)
Ischemic or hemorrhagic venous infarction	9 (3.18)	8 (4.35)	1 (1.01)	0.162	4.455 (0.549–36.140)
Thrombophilia conditions, *n* (%)					
APS	25 (8.83)	18 (9.78)	7 (7.07)	0.445	1.425 (0.574–3.539)
Protein C/S deficiency	19 (6.71)	16 (8.70)	3 (3.03)	0.083^b^	3.048 (0.866–10.726)
Essential thrombocythemia	10 (3.53)	8 (4.35)	2 (2.02)	0.323	2.205 (0.459–10.588)
Oral contraceptive use	6 (2.12)	3 (1.63)	3 (3.03)	0.443	0.530 (0.105–2.678)
Tumor	5 (1.77)	2 (1.09)	3 (3.03)	0.257	0.352 (0.058–2.141)
Pregnancy or puerperium	3 (1.06)	2 (1.09)	1 (1.01)	0.952	1.077 (0.096–12.026)
Lifestyle habits, *n* (%)					
Smoking	68 (24.03)	49 (26.63)	19 (19.19)	0.164	1.528 (0.841–2.778)
Alcohol drinking	77 (27.21)	55 (29.89)	22 (22.22)	0.168	1.492 (0.844–2.637)

Abbreviations: APS, antiphospholipid syndrome; BMI, body mass index; CCVT, cerebral cortical venous thrombosis; CI, confidence interval; CSF, cerebrospinal fluid; CVSS, cerebral venous sinus stenosis; CVST, cerebral venous sinus thrombosis; HBV, hepatitis B virus; IJVS, internal jugular venous stenosis; IQR, interquartile range; ONSD, optic nerve sheath diameter; OR, odds ratio; SD, standard deviation; T2DM, type‐2 diabetes mellitus.

^a^ Means *p* value < 0.05.

^b^ Means *p* value < 0.1.

### 3.2 Headache Features and Functional Burden Among IJVS Patients

Among patients with headache, the majority reported chronic (82.07%), bilateral (70.11%), generalized (85.87%), and intermittent (77.17%) pain patterns. Blunt‐type headache was the most commonly described type (36.96%). Most patients (88.59%) had no identifiable headache trigger, though 11.41% reported anxiety, fatigue, or sleep deprivation as provoking factors. Based on VAS scores, 40.22% had mild headaches (scores 1–3), and 53.26% had moderate headaches (scores 4–6). In terms of functional impact assessed by HIT‐6, 57.61% experienced some impairment in daily activities (scores 50–55) (Table 2).

**TABLE 2:** Clinical characteristics of headache in the IJVS‐headache group.

Items, *n* (%)	IJVS‐headache group (*n* = 184)
Onset	
Acute	33 (17.93)
Chronic	151 (82.07)
Laterality	
Unilateral	55 (29.89)
Bilateral	129 (70.11)
Region	
Generalized	158 (85.87)
Localized	26 (14.13)
Quality	
Blunt	68 (36.96)
Pressing	46 (25.00)
Stabbing	21 (11.41)
Pulling	19 (10.33)
Combined	30 (16.30)
Duration	
Persistent	42 (22.83)
Intermittent	142 (77.17)
Trigger factors	
With	21 (11.41)
Without	163 (88.59)
VAS scores	
Mild (1–3)	74 (40.22)
Moderate (4–6)	98 (53.26)
Severe (7–10)	12 (6.52)
HIT‐6 scores	
Little or no impact (49 or less)	32 (17.39)
Some impact (50–55)	106 (57.61)
Substantial impact (56–59)	35 (19.02)
Severe impact (60–78)	11 (5.98)

Abbreviations: HIT‐6, Headache Impact Test‐6; IJVS, internal jugular venous stenosis; VAS, visual analog scale.

### 3.3 Associated Clinical Manifestations and Comorbid Conditions

Non‐thrombotic IJVS patients typically presented with diverse and non‐specific symptoms. Besides headache, the top three complaints were sleep disturbances (*n* = 146, 51.59%), dizziness (*n* = 136, 48.06%), and tinnitus (*n* = 131, 46.29%), showing no significant difference between groups (all *p* > 0.1). However, patients in the IJVS‐headache group more frequently reported visual disturbances (44.02% vs. 28.28%, *p* = 0.010), nausea or vomiting (32.61% vs. 21.21%, *p* = 0.045), and neck discomfort (17.93% vs. 2.02%, *p* = 0.050). By contrast, head noise was more common in the IJVS‐without‐headache group (49.49% vs. 36.41%, *p* = 0.034) (Table 1).

All participants underwent lumbar puncture, revealing significantly higher CSF opening pressure in the IJVS‐headache group [195.00 (160.00–243.75) mmH_2_O] compared to the IJVS‐without‐headache group [165.00 (145.00–200.00) mmH_2_O, *p* < 0.001]. Papilledema was more frequently observed in patients with headache (25.00% vs. 14.14%, *p* = 0.035). ONSD was also significantly wider in the IJVS‐headache group on both the right [4.09 (3.66–4.60) mm vs. 3.80 (3.50–4.30) mm, *p* = 0.004] and left sides [4.08 (3.70–4.50) mm vs. 3.75 (3.45–4.25) mm, *p* = 0.001], possibly correlating with elevated CSF pressure (Table 1).

Comorbid venous abnormalities were more prevalent in the headache group, including high jugular bulb (19.56% vs. 7.07%, *p* = 0.007), non‐thrombotic CVSS (60.87% vs. 47.47%, *p* = 0.031), CCVT (60.33% vs. 33.33%, *p* < 0.001), and CVST (23.91% vs. 13.13%, *p* = 0.033). In univariate analysis, no significant intergroup differences were observed in cerebral parenchymal lesions or vascular/metabolic comorbidities such as hypertension, T2DM, hyperlipidemia, hyperhomocysteinemia, hyperuricemia, or previous HBV infection (all *p* > 0.1) (Table 1).

Among thrombophilic conditions, antiphospholipid syndrome (APS) was most prevalent (*n* = 25, 8.83%), followed by protein C or S deficiency (*n* = 19, 6.71%) and essential thrombocythemia (*n* = 10, 3.53%). Protein C or S deficiency tended to be more common in the IJVS‐headache group than in the IJVS‐without‐headache group (8.70% vs. 3.03%, *p* = 0.083), whereas other thrombophilic disorders did not differ significantly between groups (Table 1).

### 3.4 Imaging Characteristics of IJVS and Their Association With Headache Presentation

Of all patients, 127 (44.88%) had unilateral IJVS and 156 (55.12%) had bilateral involvement. Compared with unilateral IJVS, bilateral IJVS conferred a significantly higher risk of headache (OR = 2.210, 95% CI: 1.344–3.635, *p* = 0.002). The most frequently affected stenotic segment was J3 (83.04%), followed by J2 (15.55%) and J1 (1.41%), with no significant differences between groups (Table 3).

**TABLE 3:** Radiological features and treatment in non‐thrombotic IJVS patients with or without headache.

Variables, *n* (%)	All (*n* = 283)	IJVS‐headache group (*n* = 184)	IJVS‐without‐headache group (*n* = 99)	Univariate analysis	
				*p* value	OR (95% CI)
Stenotic side of IJV					
Unilateral IJVS	127 (44.88)	70 (38.04)	57 (57.58)	Reference	
Bilateral IJVS	156 (55.12)	114 (61.96)	42 (42.42)	0.002^a^	2.210 (1.344–3.635)
Stenotic segment of IJV					
Segment J1	4 (1.41)	2 (1.09)	2 (2.02)	0.532	0.533 (0.074–3.842)
Segment J2	44 (15.55)	28 (15.22)	16 (16.16)	0.834	0.931 (0.477–1.819)
Segment J3	235 (83.04)	154 (83.69)	81 (81.82)	0.688	1.141 (0.600–2.171)
Stenotic types					
Osseous compression	164 (57.95)	123 (66.85)	41 (41.42)	< 0.001^a^	2.852 (1.723–4.722)
Arterial compression	63 (22.26)	36 (19.56)	27 (27.27)	0.139	0.649 (0.366–1.150)
Elongated venous valves	48 (16.96)	20 (10.87)	28 (28.28)	< 0.001^a^	0.309 (0.163–0.585)
Surrounding muscle or lymph nodes compression	8 (2.83)	5 (2.72)	3 (3.03)	0.880	0.894 (0.209–3.821)
Intracranial collaterals					
No dilation	87 (30.74)	44 (23.91)	43 (43.44)	Reference	
Mild scalp vein dilation	65 (22.97)	33 (17.93)	32 (32.32)	0.981	1.008 (0.530–1.917)
Severe scalp vein dilation	131 (46.29)	107 (58.16)	24 (24.24)	< 0.001^a^	4.357 (2.366–8.022)
Extracranial collaterals					
Mild vertebral vein expansion	173 (61.13)	134 (72.83)	39 (39.39)	Reference	
Severe vertebral vein expansion	110 (38.87)	50 (27.17)	60 (60.61)	< 0.001^a^	0.243 (0.145–0.407)
Treatment					
Anticoagulation	203 (71.73)	138 (75.00)	65 (65.66)	0.097^b^	1.569 (0.921–2.672)
Stenting and/or balloon angioplasty	10 (3.53)	7 (3.80)	3 (3.03)	0.737	1.266 (0.320–5.006)
Symptomatic treatment	70 (24.74)	39 (21.20)	31 (31.31)	0.061^b^	0.590 (0.339–1.025)

Abbreviations: CI, confidence interval; IJV, internal jugular vein; IJVS, internal jugular venous stenosis; OR, odds ratio.

^a^ Means *p*‐value < 0.05.

^b^ Means *p*‐value < 0.1.

External compression was the predominant etiology of stenosis, most often caused by osseous structures (57.95%), arteries (22.26%), and surrounding muscles or lymph nodes (2.83%). Only 16.96% of patients had stenosis due to intraluminal anomalies (e.g., elongated venous valves). Osseous compression was significantly more prevalent in the IJVS‐headache group (66.85% vs. 41.42%, *p* < 0.001), while elongated venous valves were more frequent in the IJVS‐without‐headache group (28.28% vs. 10.87%, *p* < 0.001) (Table 3).

Collateral venous drainage was evaluated using CE‐MRV or CTV. In the IJVS‐headache group, scalp vein dilation was classified as severe in 58.16%, mild in 17.93%, and absent in 23.91%. In contrast, the IJVS‐without‐headache group showed a higher prevalence of absent dilation (43.44%) and a lower rate of severe dilation (24.24%). Univariate logistic regression showed that severe scalp vein dilation increased the risk of headache by 4.357‐fold (95% CI: 2.366–8.022, *p* < 0.001) relative to no dilation, whereas mild dilation did not differ from no dilation (*p* = 0.981). All patients exhibited vertebral venous collaterals surrounding the stenotic segments, although the degree varied. Among headache patients, 72.83% had mild and 27.17% had severe vertebral vein expansion; the pattern was reversed in the IJVS‐without‐headache group (39.39% mild vs. 60.61% severe). Univariate analysis showed that compared with mild vertebral vein expansion, severe expansion protected against headache (OR = 0.243, 95% CI: 0.145–0.407, *p* < 0.001) (Table 3).

### 3.5 Treatment Patterns

Overall, anticoagulation was the primary treatment, more frequently administered in the IJVS‐headache group than in the IJVS‐without‐headache group (75.00% vs. 65.66%, *p* = 0.097). Symptomatic therapies (e.g., analgesics, hypnotics, antidepressants, and anxiolytics) were more often used in patients without headache (31.31% vs. 21.20%, *p* = 0.061). Only 10 patients (3.53%) underwent endovascular procedures, including IJV stenting and/or balloon angioplasty (Table 3).

### 3.6 Independent Risk Factors for Headache in Non‐Thrombotic IJVS: A Multivariable Regression Analysis

Variables significantly different between the IJVS‐headache and IJVS‐without‐headache groups in univariate analysis (*p* < 0.1) were entered into multivariate logistic regression. Patient age and the clinical symptoms of head noise, visual disturbances, and nausea or vomiting were not independently associated with headache (*p* > 0.05). Neck discomfort, however, remained significantly associated (OR = 4.439, 95% CI: 1.470–13.408, *p* = 0.008). Although elevated CSF opening pressure, papilledema, and ONSD were more common in headache patients, these variables did not retain independent significance in the multivariate model (all *p* > 0.05).

Factors independently linked to headache included high jugular bulb (OR = 3.144, 95% CI: 1.083–9.123, *p* = 0.035), CCVT (OR = 2.446, 95% CI: 1.198–4.994, *p* = 0.014), protein C or S deficiency (OR = 5.984, 95% CI: 1.196–29.928, *p* = 0.029), bilateral IJVS (OR = 2.159, 95% CI: 1.073–4.341, *p* = 0.031), and severe scalp vein dilation (OR = 2.142, 95% CI: 1.068–4.294, *p* = 0.032). Severe vertebral vein expansion was inversely related to headache occurrence (OR = 0.184, 95% CI: 0.092–0.366, *p* < 0.001). Coexisting non‐thrombotic CVSS and CVST, different etiologies of stenosis (e.g., osseous compression and intraluminal elongated venous valves), anticoagulation, and symptomatic treatments did not show an independent association with headache (all *p* > 0.05) (Table 4).

**TABLE 4:** Multivariate logistic regression analysis in non‐thrombotic IJVS patients.

Variables	*p* value	OR (95% CI)
Age	0.057	0.975 (0.951–1.001)
Head noise	0.819	0.921 (0.455–1.863)
Visual disturbances	0.199	1.606 (0.780–3.306)
Nausea or vomiting	0.511	1.298 (0.596–2.828)
Neck discomfort	0.008^a^	4.439 (1.470–13.408)
CSF opening pressure	0.101	1.006 (0.999–1.014)
Papilledema	0.854	0.900 (0.294–2.755)
Right ONSD	0.176	0.431 (0.127–1.459)
Left ONSD	0.120	2.570 (0.782–8.452)
High jugular bulb	0.035^a^	3.144 (1.083–9.123)
Non‐thrombotic CVSS	0.198	1.588 (0.785–3.211)
CCVT	0.014^a^	2.446 (1.198–4.994)
CVST	0.676	1.233 (0.462–3.291)
Protein C/S deficiency	0.029^a^	5.984 (1.196–29.928)
Bilateral IJVS	0.031^a^	2.159 (1.073–4.341)
Osseous compression	0.053	2.145 (0.992–4.640)
Elongated venous valves	0.477	0.703 (0.266–1.858)
Severe scalp vein dilation	0.032^a^	2.142 (1.068–4.294)
Severe vertebral vein expansion	< 0.001^a^	0.184 (0.092–0.366)
Anticoagulation	0.651	1.528 (0.243–9.599)
Symptomatic treatments	0.451	2.094 (0.307–14.273)

Abbreviations: CCVT, cerebral cortical venous thrombosis; CI, confidence interval; CSF, cerebrospinal fluid; CVSS, cerebral venous sinus stenosis; CVST, cerebral venous sinus thrombosis; IJVS, internal jugular venous stenosis; ONSD, optic nerve sheath diameter; OR, odds ratio.

^a^ Means *p* value < 0.05.

## 4 Discussion

### 4.1 Headache and Venous Outflow Obstruction in Non‐Thrombotic IJVS

IJVS is defined as ≥ 50% narrowing of the IJV lumen relative to an adjacent normal segment, typically accompanied by dilated paraspinal venous collaterals on imaging. Non‐thrombotic IJVS refers to stenosis in the absence of thrombus formation [2, 11]. Its clinical presentation is often nonspecific, encompassing tinnitus, head noise, visual impairment, sleep disturbances, neck discomfort, and memory decline [1, 2, 3, 4, 5, 6, 7]. Among these symptoms, headache is a common but frequently underrecognized neurological complaint in non‐thrombotic IJVS patients [1, 2, 3, 4, 5, 6, 7, 9].

Emerging evidence links IJVS‐associated headache to impaired cerebral venous drainage. As the primary extracranial pathway for cerebral venous outflow, IJVS may lead to intracranial venous hypertension, venous congestion, and dural sinus dilation—all of which can activate the trigeminovascular system and trigger headache [9, 12, 15, 16, 17, 18, 19]. Given the significant impact of headache on patients' quality of life, this study sought to characterize its clinical features and identify associated risk factors in non‐thrombotic IJVS.

### 4.2 Clinical Characteristics of Non‐Thrombotic IJVS With Headache

Our univariate analysis revealed that non‐thrombotic IJVS patients with headache were more likely to experience visual disturbances, nausea or vomiting, neck discomfort, papilledema, and bilateral ONSD widening. These clinical features were largely associated with elevated CSF opening pressure. IJVS increases resistance to cerebral venous outflow, resulting in impaired CSF reabsorption through the venous system, ultimately contributing to raised CSF pressure [3, 4, 20, 21, 22]. This resulting pressure elevation can further compress brain tissue and stretch pain‐sensitive structures, causing headache and associated symptoms [1, 3, 6, 11]. The chronic, bilateral, and generalized headache pattern observed in IJVS may reflect sustained cephalocervical venous congestion and global cerebral hypoperfusion [1, 11]. Anatomically, the optic nerve sheath is a continuation of the dura mater and communicates freely with the intracranial subarachnoid space, allowing CSF to surround the optic nerve along its length. Elevated CSF pressure can distend this sheath and exert compressive effects on the optic nerve, leading to papilledema and visual disorders such as diplopia, blurred vision or visual field defects [23, 24, 25, 26, 27, 28, 29].

Multivariate analysis further identified neck discomfort as an independent predictor of headache in non‐thrombotic IJVS patients. This association may be explained by the anatomical convergence of upper cervical spinal nerves (C1–C3) with the trigeminocervical complex within the upper spinal cord. Nociceptive input from cervical structures—such as muscles, ligaments, or joints—may stimulate the trigeminal system through the trigeminal spinal tract, thereby triggering cervicogenic headache [30, 31, 32, 33].

Of particular note, our study also found protein C or S deficiency to be independently associated with headache. These thrombophilic states promote hypercoagulability and may result in subclinical microthrombi or endothelial dysfunction. Such changes could initiate cortical spreading depression and neuroinflammatory cascades, which are known to activate both peripheral and central components of the trigeminovascular system, ultimately contributing to headache pathogenesis [34, 35].

### 4.3 Neuroimaging Insights and Hemodynamic Mechanisms

Non‐thrombotic IJVS can arise from either extraluminal compression or intraluminal anomalies, as revealed by ultrasonography, CTV, or CE‐MRV. Owing to its thin wall and lack of smooth muscle, the IJV is highly susceptible to external compression from adjacent anatomical structures, including bony abnormalities, the carotid artery, hypertrophied muscles, enlarged lymph nodes, or the thyroid gland. Among these, osseous impingement—particularly from elongated C1 transverse processes or a prominent styloid process—has been identified as a major contributor to IJV narrowing [1, 2, 3, 4, 5, 36, 37, 38, 39]. In our study, over half of the headache group exhibited osseous compression, highlighting its clinical relevance. Intraluminal causes of stenosis include malformed venous valves, fibrotic wall lesions, or thrombosis [1, 2, 3, 4]. Cases with IJVT were excluded from this study to ensure the focus remained on non‐thrombotic etiologies. Regardless of the cause, impaired IJV patency compromises cerebral venous drainage and increases intracranial venous pressure [3, 5, 6]. As a compensatory response, many patients develop collateral circulation, most notably via dilated scalp veins and engorged vertebral venous plexuses. These alternative pathways serve to bypass the obstructed IJV segment and reduce outflow resistance [2, 3, 4, 7, 40]. In this study, all patients with headache exhibited vertebral vein expansion, and the majority showed varying degrees of scalp vein dilation.

**FIGURE 2:**
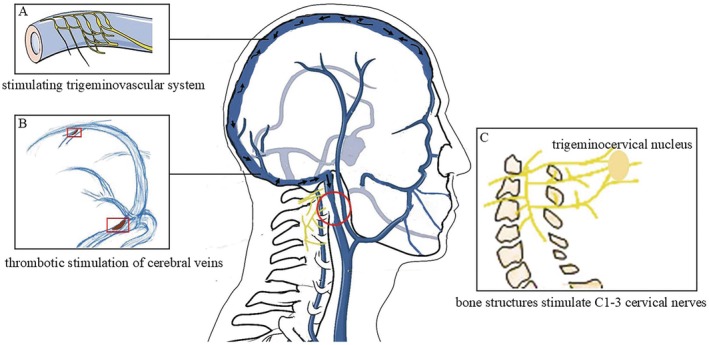
Mechanisms underlying headache in non‐thrombotic IJVS patients: (A) Stenotic IJV (in red circle box) impedes intracranial venous drainage, leading to cerebral venous congestion, which stretches pain‐sensitive structures and activates the trigeminovascular system, resulting in headache; (B) Direct thrombosis stimulation of dural sinuses and cerebral cortical veins (in red rectangle boxes) results in headache in non‐thrombotic IJVS patients with CVST and CCVT; (C) Abnormal bone structures impinging the IJV may simultaneously stimulate C1‐3 cervical spinal nerves, triggering headache via the trigeminocervical nucleus. CCVT, cerebral cortical venous thrombosis; CVST, cerebral venous sinus thrombosis; IJV, internal jugular vein; IJVS, internal jugular vein stenosis.

Patients with headache also had a higher prevalence of other venous anomalies, including high jugular bulb, non‐thrombotic CVSS, CCVT, CVST, bilateral IJVS, osseous compression, and severe scalp vein dilation. These findings suggest a greater burden of cerebral venous outflow obstruction in this subgroup. Non‐thrombotic CVSS and CVST directly impede intracranial venous drainage, while high jugular bulb and bilateral IJVS further compromise extracranial venous outflow, leading to cerebral venous engorgement and scalp vein dilation. This mechanical stress may distend dural sinus walls and stimulate perivascular nociceptive fibers, thereby activating the trigeminovascular system and eliciting headache (Figure 2A) [3, 5, 20, 21, 36, 39, 41, 42, 43, 44]. Moreover, direct thrombotic involvement of dural sinuses and cortical veins in CVST and CCVT may aggravate venous hypertension and further provoke nociceptor activation (Figure 2B) [3, 5, 11, 12, 39]. Another underlying mechanism of headache is the impingement of the IJV by abnormal bone structures, which may concurrently stimulate C1–C3 cervical spinal nerves and then initiate cervicogenic headache through the trigeminocervical complex (Figure 2C) [30].

Interestingly, some imaging findings were inversely associated with headache. Patients with elongated venous valves—a relatively benign intraluminal anomaly—showed a lower incidence of headache. These valves may permit more efficient formation of intraluminal or paravenous collaterals, maintaining venous outflow. Likewise, severe vertebral vein expansion appeared to offer a protective effect by enhancing collateral drainage capacity, reducing venous congestion, and mitigating headache severity [2].

### 4.4 Therapeutic Implications and Future Directions

The management of headache in non‐thrombotic IJVS remains clinically challenging due to its heterogeneous presentations and the absence of standardized treatment guidelines. Current therapeutic options—ranging from anticoagulation and symptomatic control to endovascular intervention—must be tailored based on individual risk profiles and underlying pathophysiology [4, 11, 36, 39].

In this study, anticoagulation was more frequently administered in patients with headache, likely due to the higher prevalence of coexisting CVSS, CCVT, CVST, and thrombophilic conditions (particularly protein C or S deficiency). Given the well‐established link between hypercoagulability and cerebral venous thrombosis, early anticoagulation may offer clinical benefits in selected patients to prevent progression and alleviate headache symptoms [3, 11, 45]. However, its utility in patients without overt thrombosis remains uncertain, and robust evidence from prospective studies is needed to assess the long‐term efficacy and safety of anticoagulation in purely stenotic, non‐thrombotic IJVS.

Supportive symptomatic therapies—including analgesics, anti‐inflammatory agents, and neuromodulators (e.g., tricyclic antidepressants, topiramate)—may offer partial relief, particularly in patients with central sensitization [20, 30, 33, 41]. Nonetheless, their effectiveness is often limited in cases of persistent venous congestion or elevated intracranial venous pressure, highlighting the need for etiologically targeted interventions in selected patients.

Endovascular treatment, especially IJV stenting, has emerged as a promising option for restoring venous outflow and reducing intracranial venous hypertension in severe cases. Several studies have reported substantial improvements in headache severity and overall quality of life following stent placement in patients with significant IJVS [1, 3, 4, 7, 21, 36, 37, 46]. However, not all patients are appropriate candidates for stenting. For instance, those with well‐developed collateral pathways—particularly severe vertebral vein expansion—may achieve adequate compensatory drainage and derive limited benefit from invasive procedures. Thus, future research should prioritize individualized hemodynamic evaluation, including assessments of jugular outflow resistance and collateral efficiency, to refine patient selection criteria.

Well‐designed prospective randomized trials are essential to determine whether endovascular therapy offers superior outcomes compared to conservative approaches in high‐risk subgroups. Moreover, long‐term follow‐up studies are needed to evaluate the durability of symptom relief and identify predictors of treatment response. By adopting a personalized, risk‐stratified therapeutic framework, clinicians may enhance symptom control and neurological outcomes in patients with IJVS‐related headache, ultimately guiding more targeted and effective clinical decision‐making.

## 5 Limitations

This study has several limitations that warrant consideration. First, its single‐center retrospective design may introduce selection bias, as the enrolled patient population may not fully represent the broader demographic and clinical heterogeneity observed in real‐world settings. Consequently, the generalizability of our findings may be limited. To corroborate and extend these results, large‐scale, multicenter prospective studies involving diverse populations and care environments are necessary. Second, the subjective nature of headache perception and reporting may lead to misclassification or variability in symptom assessment, potentially influencing group stratification and outcome interpretation. Third, there is currently no standardized or validated method for quantifying scalp vein dilation. Our assessment relied on visual inspection, which, despite being performed by experienced clinicians, may introduce interobserver variability and reduce measurement precision. Future research should aim to incorporate objective imaging‐based criteria for vascular evaluation and standardized headache classification systems to improve reproducibility and diagnostic accuracy.

## 6 Conclusions

Headache is a prevalent manifestation in patients with non‐thrombotic IJVS, typically presenting as chronic, diffuse, and moderate in intensity. Bilateral IJVS, high‐positioned jugular bulb, CCVT, severe scalp vein dilation, and protein C or S deficiency were independently associated with an increased risk of headache, whereas pronounced vertebral venous expansion appeared protective. These findings offer both clinical and radiological evidence implicating cerebral venous outflow obstruction in the pathophysiology of headache among non‐thrombotic IJVS patients. For this patient population, comprehensive diagnostic workups—particularly neuroimaging assessments and thrombophilia screening—are essential to elucidate underlying mechanisms and guide individualized therapeutic strategies. Early identification of high‐risk features may facilitate more effective symptom control and improve overall neurological outcomes in the context of IJVS‐related headache.

## Author Contributions

R.M. and D.Z.: manuscript drafting and revision, study concept and design, and final approval of the manuscript. G.H.: manuscript drafting, data interpretation, and figure preparation. S.W. and X.J.: data collection.

## Ethics Statement

Approval was obtained from the ethics committee of Xuanwu Hospital, Capital Medical University (LYS[2022]029). The procedures used in this study adhere to the tenets of the Declaration of Helsinki.

## Consent

Written informed consent for publication was obtained from all participants involved in the study.

## Conflicts of Interest

The authors declare no conflicts of interest.

## Data Availability

The data that support the findings of this study are available from the corresponding author upon reasonable request.
